# Supplementary material for: Determinants of In-Hospital Mortality in Elderly Patients Aged 80 Years or above with Acute Heart Failure: A Retrospective Cohort Study at a Single Rural Hospital
Source: J Clin Med. 2021 Apr 2;10(7):1468. doi: 10.3390/jcm10071468 (PMC8038161; doi:10.3390/jcm10071468)
Supplement: Supplementary file 1 [file jcm-10-01468-s001.pdf]

Supplemental Table S1. Univariate logistic regression analysis to predict in-hospital mortality

Univariate Analysis	OR	95 % CI	*P*-value
Baseline characteristics at admission			
Height (per cm increase)	1.024	0.975–1.075	0.34
Body weight (per kg increase)	0.968	0.927–1.016	0.19
Diastolic blood pressure (per mmHg increase)	0.985	0.962–1.008	0.20
Heart rate (per beats/minute increase)	1.006	0.989–1.022	0.51
Respiratory rate (per breaths/minute)	0.914	0.810–1.030	0.14
Hypertension	0.967	0.187–5.006	0.97
Dyslipidemia	0.820	0.271–2.482	0.73
Diabetes mellitus	0.819	0.289–2.322	0.71
Atrial fibrillation/atrial flutter	1.839	0.684–4.938	0.23
Pacemaker implantation	0.245	0.030–1.978	0.19
Chronic obstructive pulmonary disease	1.034	0.200–5.352	0.97
eGFR < 60 mL/min/1.73 m^2	0.824	0.299–2.270	0.71
Ambulance transport to emergency department	1.478	0.503–4.346	0.48
NYHA functional classification grade 4 at admission (vs. 3)	1.736	0.582–5.173	0.32
Medication at admission			
ACE-I and/or ARB	0.492	0.192–1.265	0.14
β blockers	0.907	0.334–2.466	0.85
Calcium channel blockers	0.600	0.223–1.614	0.31
Loop diuretics	2.170	0.777–6.060	0.14
Thiazides	3.857	0.513–29.015	0.19
Tolvaptan	1.925	0.442–8.377	0.38
Digitalis	0.898	0.095–8.447	0.93
Statins	0.888	0.228–3.454	0.86
Oral anti-diabetes mellitus agents	0.563	0.117–2.720	0.48
Anti-platelets	1.926	0.705–5.258	0.20
Anti-coagulants	0.576	0.193–1.717	0.32
Laboratory data			
Total protein (per g/dL increase)	0.69	0.360–1.325	0.27
Total bilirubin (per g/dL increase)	0.875	0.321–2.380	0.79
Blood glucose (per mg/dL increase)	1.005	0.996–1.015	0.29
Blood urea nitrogen (per mg/dL increase)	1.009	0.983–1.036	0.50
Serum creatinine (per mg/dL increase)	1.024	0.542–1.934	0.94
Estimated glomerular filtration rate (per mL/min/1.73 m^2 increase)	0.992	0.971–1.014	0.47
Hemoglobin (per g/dL increase)	1.03	0.810–1.310	0.81
Echocardiography parameters			
Interventricular septal thickness (per mm increase)	0.983	0.770–1.255	0.89

Left ventricular end-diastolic diameter (per mm increase)	**1.008**	**0.960–1.058**	**0.75**
Left ventricular end-systolic diameter (per mm increase)	**1.037**	**0.992–1.084**	**0.11**
Posterior left ventricular wall thickness (per mm increase)	1.063	0.834–1.354	0.62
Left ventricular end-diastolic volume (per mL increase)	**1.003**	**0.994–1.013**	**0.49**
**Left ventricular ejection fraction (per % increase)**	0.968	0.943–0.994	0.016
Left atrial diameter (per mm increase)	0.985	0.934–1.038	0.57
Valvular heart disease	2.678	0.321–22.294	0.36
Wall motion abnormality	1.731	0.481–6.233	0.40
Treatment within 48 h of hospitalization			
Intravenous carperitide administration	N/A	N/A	N/A
Tolvaptan introduction	N/A	N/A	N/A
Intravenous nitric acid administration	0.298	0.036–2.435	0.26
Digoxin administration	2.540	0.398–16.194	0.32
PDE3-inhibitor and/or catecholamine addition	N/A	N/A	N/A
NPPV support requirement	2.962	0.613–14.315	0.18
Morphine use, n	N/A	N/A	N/A
Treatment and results during the entire period of hospitalization			
Intravenous diuretic administration	0.794	0.255–2.477	0.69
Intravenous carperitide administration	N/A	N/A	N/A
Tolvaptan introduction	2.576	0.823–8.068	0.10
Intravenous nitric acid administration	0.298	0.036–2.435	0.26
Digoxin administration	1.881	0.322–10.980	0.48
Intravenous catecholamine support requirement	11.667	2.723–49.992	<0.001
PDE3-inhibitor and/or catecholamine addition	3.857	0.513–29.015	0.19
Intravenous antibiotics administration	4.568	1.709–12.211	0.0025
NPPV support requirement	4.333	1.133–16.572	0.032
Morphine use	3.857	0.513–29.015	0.19
Maximum serum creatinine during hospitalization (per mg/dL increase)	1.645	1.072–2.524	0.023
Worsening renal function	6.514	2.398–17.695	<0.001

Categorical variables are expressed as numbers. ACE-I: angiotensin-converting enzyme inhibitor; ARB: angiotensin II receptor blocker; CI: confidence interval; eGFR: estimated glomerular filtration rate; NPPV: noninvasive positive pressure ventilation; NYHA: New York Heart Association; OR: odds ratio; PDE3: phosphodiesterase 3.
